# Expression of transient receptor potential channel vanilloid (TRPV) 1–4, melastin (TRPM) 5 and 8, and ankyrin (TRPA1) in the normal and methimazole-treated mouse olfactory epithelium

**DOI:** 10.3109/00016489.2010.489573

**Published:** 2010-06-30

**Authors:** Yousuke Nakashimo, Masaya Takumida, Takashi Fukuiri, Matti Anniko, Katsuhiro Hirakawa

**Affiliations:** 1 Department of Otolaryngology, Hiroshima University Faculty of Medicine, Hiroshima, Japan; 2 Department of Otolaryngology, Head and Neck Surgery, University Hospital, Uppsala, Sweden

**Keywords:** Immunohistochemistry, olfactory chemosensation, olfactory disturbance

## Abstract

*Conclusion:* It is suggested that TRPV1, 2, 3, and 4, TRPM5 and 8, and TRPA1 may play several roles in the olfactory epithelium *(OE),* contributing to olfactory chemosensation, olfactory adaptation, olfactory-trigeminal interaction, and OE fluid homeostasis. In patients with olfactory disturbance, TRPV1 and TRPM8 may be closely related to a high rate of recognition of curry and menthol odors, while TRPV2 may also play a crucial role in the regeneration of olfactory receptor neurons. *Objective:* Expression of TRPV1–4, TRPM5 and 8, and TRPA1 in the normal and methimazole-treated mouse OE was analyzed. *Methods:* The localization of TRPV1–4, TRPM5 and 8, and TRPA1 in the OE of normal and methimazole-treated CBA/J mice was investigated by immunohistochemistry. *Results:* Normal OE showed a positive immunofluorescent reaction to TRPV1–4, TRPM5 and 8, and TRPA1. In lamina propria, the nerve fibers displayed TRPV 1, 2, and 3, TRPM8 and TRPA1. In the pathological condition, the expression of TRPV3, TRPV4, TRPM5, and TRPA1 was markedly reduced and took a long time to recover. In contrast, expression of TRPM8 was scarcely affected, even in the pathological condition, while TRPV1 and TRPV2 showed early recovery following methimazole treatment.

## Introduction

The TRP (transient receptor potential) ion channel family comprises 28 channels, divided into 6 subgroups according to their structure and activation characteristics. TRP subfamilies include canonical (TRPC, seven channels), melastatin (TRPM, eight), ankyrin (TRPA, one), vanilloid (TRPV, six), poly-cystin (TRPP, three), and mucolipin (TRPML, three channels). TRP channels play a crucial role in the responses to all major classes of external stimuli, including light, sound, chemicals, temperature, and touch. TRP channels also imbue individual cells with the ability to sense changes in the local environment, such as alterations in osmolality [1].

Thermosensing can be considered the most elementary of all senses, as it is absolutely crucial for our survival. A prompt reaction to contact with harmfully cold or hot objects is vital to prevent acute, potentially fatal, injury. Moreover, to maintain the core body temperature of around 37°C, heat production and heat loss must be maintained equal in steady state. This requires the permanent monitoring and integration of thermal information from the skin (via peripheral thermoreceptors) and deep body structures (via central thermoreceptors), and the ensuing initiation of reflexes that promote heat production or heat loss.

Recent studies have provided the first molecular insight into the mechanisms underlying the exquisite thermo- and chemosensitivity of these channels. Moreover, accumulating evidence implicates TRP channels in the development of the central nervous system. A superfamily subset of TRPs, dubbed thermo-TRPs, are highly sensitive to temperature, and several of them serve essentially as molecular thermometers in different cell populations of the peripheral sensory system [2].

In the olfactory epithelium (OE), expression of several TRP channels has been reported. Recent investigations revealed the expression of TRPVs and a possible functional role in the OE [3,4], TRPV1-4 variably with a diffuse pattern in lamina propria, and in the respiratory epithelium, especially so in glandular cells of lamina propria. These findings suggested that TRPVs may play a variety of roles in the OE, contributing to olfactory adaptation, olfac-tory-trigeminal interactions in nasal chemoreception, and OE homeostasis; they may also be involved in olfactory transduction as well as olfactory dysfunction secondary to sinonasal inflammation disease [3]. However, the detailed distribution of TRPVs in the OE and the expression of other TRP channels in the OE, especially in pathological conditions, is still not known.

In this investigation we identified TRPV1-4 as well as TRPM5 and 8 and also TRPA1 in the OE in both normal and methimazole-treated mice. As these TRP channels are regarded as thermo-TRPs, it has recently been shown that temperature can influence olfaction [5].

## Material and methods

We used 15 healthy, normal, 8-week-old CBA/J mice with body weights in the range 20-25 g. They were housed in a light-controlled room with a 12 h light/ dark cycle and were allowed access to food and water ad libitum. Care and use of the animals was approved by the Animal Experimentation Committee, Hiroshima University School of Medicine (permit no. A08-88) and was in accordance with the Guide to Animal Experimentation, Hiroshima University and the guidelines of the Committee on Research Facilities for Laboratory Animal Science, Hiroshima University School of Medicine.

For the methimazole-treated study, 12 animals were injected intraperitoneally (i.p.) with 300 mg/ kg methimazole dissolved in 2 ml of 0.01 M phosphate-buffered saline (PBS) containing 20% dimethyl sulfoxide (DMSO). These animals were used for the immunohistochemical study 1 day, 7 days, 4 weeks, and 3 months after the injection. The normal and methimazole-treated animals were deeply anesthetized with pentobarbital and fixed by cardiac perfusion with 4% paraformaldehyde in 0.1 M phosphate buffer solution, pH 7.4. Nasal tissue, including the OE, was excised and immersed in the same fixative for a further 1 h. It was decalcified with 0.1 M buffered Na-EDTA for 14 days. The specimens were cryoprotected in cold 20% sucrose in PBS, frozen in OCT mounting medium (Sakura Finetech-nical Co. Ltd, Tokyo, Japan). Coronal sections were serially cut on a cryostat at 4 mm, and mounted on glass slides. After pretreatment with blocking serum, the specimens were incubated with a rabbit polyclonal antibody to TRPV1 (Transgenic Inc., Kumamoto, Japan) (diluted 0.1 mg/ml), rabbit polyclonal antibody to TRPV2 (abcam, Tokyo, Japan) (diluted 1:1000), goat polyclonal antibody to TRPV3 (Santa Cruz Biotechnology Inc, CA, USA) (diluted 1:50), a rabbit polyclonal antibody to TRPV4 (Alomone Labs Ltd, Jerusalem, Israel) (diluted 1:200), a goat polyclonal antibody to TRPM5 (Santa Cruz Biotechnology) (diluted 1:200), a rabbit polyclonal antibody to TRPM8 (Osenses Ltd) (diluted 1:300), or with a rabbit polyclonal antibody to TRPA1 (Transgenic) (diluted 1:200) in 0.3% Triton X-100-containing PBS at 4°C for 48 h. The specimens were then washed in PBS and incubated for 1 h with Alexa Fluor 488 goat anti-rabbit or donkey anti-goat secondary antibodies (1:500) (Molecular Probes, Eugene, OR, USA). The sections were washed and coverslipped with DakoCytomatia Fluorescent Mounting Medium (DakoCytomatia, CA, USA). The specimens were viewed in a Nikon fluorescence microscope (Eclipse E600) equipped with an appropriate filter set. Fluorescence analog images were obtained via an intensified digital color charge-coupled device camera (C4742-95; Hamamatsu Photonics) and stored as digital images using IP Lab Spectrum software (version 3.0; Signal Analytics Corp.). Appropriate controls with omission of the primary antibodies, or with specific blocking peptides of the primary antibodies, were included in the immunohistochemical protocol.

### Measurement of fluorescence intensity

For the statistical analysis, we measured the fluorescence intensity in the olfactory receptor neurons (ORNs). ORNs were randomly selected from each specimen, and their fluorescence intensity was determined. The value of 10 cells was averaged for each specimen. The grand mean was obtained by averaging the means of six to eight specimens for normal and methimazole-treated animals. A standard error was calculated from the means for individual specimens. These data were analyzed by a two-way analysis of variance (ANOVA).

## Results

### Distribution of TRPV1-4, TRPM5, TRPM8, and TRPA1 in mouse OE

Under light microscopy, supporting cells, ORNs, and basal cells were readily identified by the shape and location of their nuclei. In lamina propria, nerve fibers, vessels, and Bowman's glands were observed.

*TRPV1.* In the OE, an immunofluorescent reaction to TRPV1 was observed in the ORNs, especially in dendrites and axons. Supporting cells and basal cells showed a weaker reaction to TRPV1. In lamina propria, nerve fibers and blood vessel walls were immunofluorescent.

*TRPV2.* An immunofluorescent reaction to TRPV2 was observed in the ORNs, especially in dendrites and axons. Basal cells were noticeably immunofluorescent, but supporting cells only weakly so. In lamina propria, nerve fibers were markedly immunofluorescent, while blood vessel walls and Bowman's glands were only moderately so.

*TRPV3.* An immunofluorescent reaction to TRPV3 was observed in the ORNs. Supporting cells and basal cells showed weaker immunofluorescence. In lamina propria, nerve fibers and blood vessel walls were weakly immunofluorescent.

*TRPV4.* An immunofluorescent reaction to TRPV4 was observed in the ORNs. Supporting cells and basal cells showed weak immunofluorescence to TRPV1. In lamina propria, TRPV4 labeling was comparatively weak and was absent altogether in nerve fibers.

*TRPA1.* The ORNs showed an immunofluorescent reaction to TRPA1. In lamina propria, Bowman's glands showed marked immunofluorescence, while the nerve fibers showed faint fluorescence.

*TRPM5.* ORNs were moderately immunofluorescent to TRPM5 and olfactory cilia were intensely immunofluorescent, while in the lamina propria, TRPM5 labeling was comparatively weak.

*TRPM8.* ORNs were intensely immunofluorescent to TRPM8 and olfactory cilia showed moderate immunofluorescence. Basal cells were also moderately immunofluorescent, but supporting cells revealed no fluorescence at all. In the lamina propria, nerve fibers showed marked immunofluorescence.

**Figure 1:**
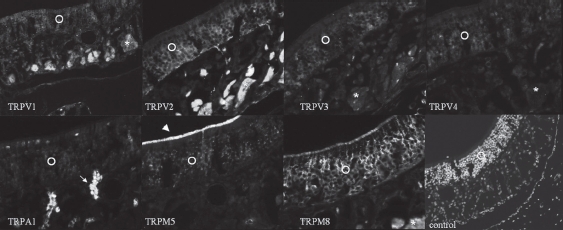
Immunofluorescent reaction to TRPV1 is evident in olfactory receptor neurons (ORNs). Supporting cells and basal cells are less intensely immunofluorescent. In lamina propria, nerve fibers (NFs) are immunofluorescent (TRPV1). Immunofluorescence to TRPV2 is evident in ORNs. Basal cells are markedly immunofluorescent. NFs are intensely immunofluorescent (TRPV2). Immunofluorescence to TRPV3 is observed in the ORNs. NFs show weak immunofluorescence (TRPV3). Immunofluorescence to TRPV4 is observed in the ORNs. In the lamina propria, TRPV4 labeling is weak and absent in NFs (TRPV4). The ORNs show immunofluorescence to TRPA1. Bowman's glands show marked immunofluorescence (TRPA1). Moderate immunofluorescence to TRPM5 is noted in the ORNs. Olfactory cilia are intensely immunofluorescent (TRPM5). Intense immunofluorescence to TRPM8 is observed in the ORNs. Olfactory cilia and basal cells are moderately immunofluorescent. NFs show marked immunofluorescence (TRPM8). Control staining without primary antibodies does not elicit immunofluorescence. Nuclei are counterstained with DAPI (control). O, ORNs; asterisk, nerve fiber; arrow, Bowman's gland; arrowhead, olfactory cilia.

*Contol.* Control staining without primary antibodies -or with specific blocking peptides of the primary antibodies - revealed no significant immunofluorescence (Figure 1).

### Changes in expression of TRPs in methimazole-treated mouse OE

**Figure 2:**
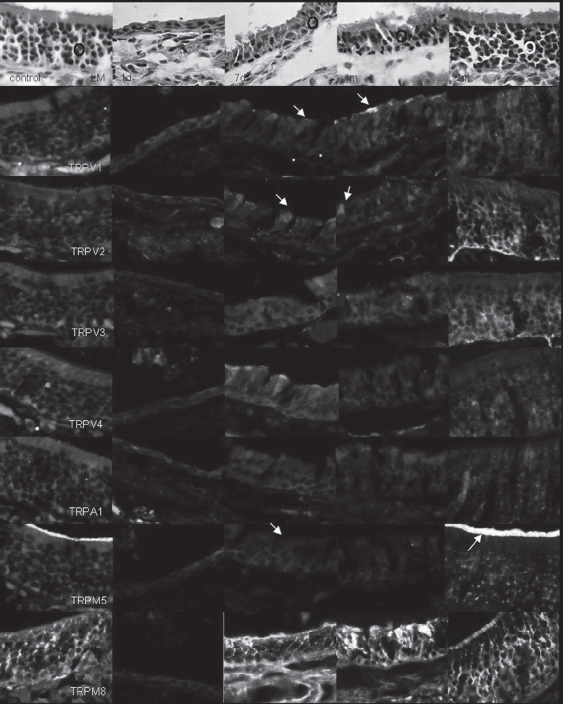
At 1 day after methimazole treatment (1d), there is a thin and disorganized neuroepithelium in the whole olfactory epithelium (OE; O). ORNs have almost completely disappeared. At 7 days later, OE shows signs of recovery but is still thin and disorganized (7d). At 1 month after treatment (1m), the OE is thicker, and after 3 months (3m) has regained its normal appearance and thickness. In general, the immunoreactivity of ORNs to TRPV1 is markedly reduced 7 days after methimazole treatment. A few cells show immunofluorescence (arrow). Immunofluorescence of ORNs has almost recovered (arrow) after 1 month and shows complete recovery after 3 months (TRPV1). Immunoreactivity of ORNs to TRPV2 is reduced in most ORNs, while a few cells show marked fluorescence (arrow) 7 days after the treatment. After 1 month, some cells show intense fluorescence (arrow), while others still show weaker fluorescence (TRPV2). Immunoreactivity of ORNs to TRPV3, TRPV4, and TRPA1 is reduced 7 days and 1 month after treatment. Immunoreactivity to TRPM5 is reduced after 7 days and 1 month. There is no fluorescence in olfactory cilia (arrow), while after 3 months, ORNs show normal fluorescence and there is intense staining in the olfactory cilia (arrow) (TRPM5). The immunoreactivity to TRPM8 is slightly weaker, but still strong after 7 days and 1 month, recovering to normal after 3 months (TRPM8).

One day after treatment with methimazole, a thin, disorganized neuroepithelium was observed throughout the olfactory region. ORNs had almost completely disappeared, remaining only in a few basal cells. One week later, OE showed signs of recovery but was still thin and disorganized. One month after the treatment, OE had become thicker and had regained its normal appearance and thickness after 3 months. In general, the immunoreactivity of ORNs to TRPV1 was markedly weaker 7 days after the methimazole treatment, although a few cells were immu-nofluorescent. The immunofluorescence of some ORNs had recovered after 1 month and showed complete recovery after 3 months. In the lamina propria, the nerve fibers were noticeably less immu-nofluorescent after 1 and 7 days and 1 month and showed normal fluorescence after 3 months. The immunoreactivity to TRPV2 was weaker in most ORNs, although a few cells showed intense fluorescence 7 days after the treatment. After 1 month, some cells were intensely fluorescent, while others still showed weaker fluorescence. After 3 months, the immunofluorescence of ORNs had returned to normal levels of intensity. In the lamina propria, the nerve fibers were markedly less immu-nofluorescent after 1 and 7 days and 1 month, but had regained normally strong fluorescence after 3 months. The immunoreactivity of ORNs to both TRPV3 and TRPV4 was weaker 7 days and 1 month after treatment, but after 3 months had returned to normal. The immunoreactivity of ORNs to TRPA1 was weaker after 7 days and 1 month, but had returned to normal after 3 months. The immunoreactivity of ORNs to TRPM5 was weaker after 7 days and 1 month but there was no fluorescence in the olfactory cilia, whereas after 3 months, fluorescence was normal in ORNs and intense in the olfactory cilia. The immunoreactivity to TRPM8 was slightly weaker, but still strong after 7 days and 1 month, and had recovered after 3 months (Figure 2).

### Fluorescence intensity (normal mice)

*TRPV1.* Immunoreactivity in normal ORNs was 62 ± 2.0 (mean ± SD), decreasing significantly (*p* < 0.01) to 30 ± 10.6 after 7 days of treatment, had almost recovered (56 ± 14.0) after 1 month, and was normal again (68 ± 8.6) after 3 months. The difference in fluorescence intensity between 7 days and 1 month after treatment was significant (*p* < 0.01).

*TRPV2.* Immunoreactivity in normal ORNs was 80 ± 25.9, decreasing significantly (*p* < 0.01) to 31 ± 10.2 after 7 days, was 48 ± 15.1 after 1 month (*p* < 0.01), and had recovered to 83 ± 17.2 after 3 months. The difference in intensity between 7 days and 1 month after treatment was significant (*p* < 0.05).

*TRPV3.* Immunoreactivity in normal ORNs was 79 ± 20.1, had decreased significantly (*p* < 0.01) after 7 days, to 51 ± 4.3 after 1 month (*p* < 0.01), and had recovered to 87 ± 14.8 after 3 months.

*TRPV4.* Immunoreactivity in normal ORNs at 79 ± 24.5 had decreased significantly 7 days later (*p* < 0.01) and to 43 ± 9.7 after 7 days (*p* < 0.01) and was 40 ± 9.5 after 1 month (*p* < 0.01), but had recovered to 76 ± 21.3 after 3 months.

*TRPA1.* Immunoreactivity in normal ORNs was 59 ± 12.6, had decreased significantly (*p* < 0.01) to 29 ± 7.7 after 7 days, was 26 ± 8.2 (*p* < 0.01) after 1 month, and had recovered to 55 ± 13.7 after 3 months.

*TRPM5.* Immunoreactivity in normal ORNs at 66 ± 24.5 had decreased significantly (*p* < 0.01) to 26 ± 7.3 after 7 days and 27 ± 6.1 after 1 month. It had recovered to 55 ± 15.5 after 3 months.

**Figure 3:**
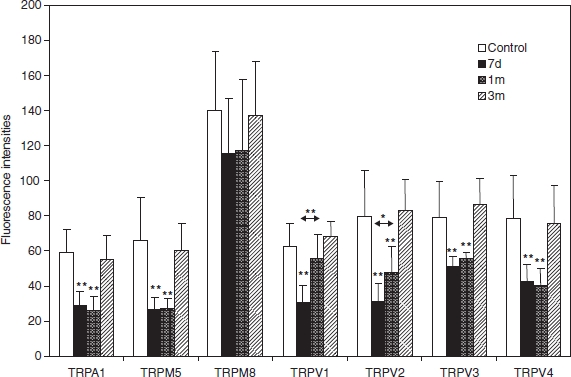
The fluorescence intensity to TRPV1 decreases significantly after 7 days but has normalized after 3 months. There is a significant difference in fluorescence intensity between 7 days and 1 month after. The intensity decreases significantly after 7 days and 1 month, with a significant difference between these two time points. The fluorescence intensities to TRPA1, TRPM5, TRPV3, and TRPV4 are significantly reduced after 1 month and 3 months, but have normalized after 3 months. The fluorescence intensity to TRPM8 is weaker but not significant after 7 days and 1 month. **p < 0.01, *p < 0.05.

*TRPM8.* Immunoreactivity in normal ORNs at 141 ± 33.2 was significantly weaker (*p* < 0.01) at 115 ± 31.8 after 7 days and 117 ± 40.6 after 1 month (*p* < 0.1) but had normalized to 138 ± 30.1 after 3 months (Figure 3).

## Discussion

There is accumulating evidence that TRP channels are involved in thermosensation, mechanosensation, smell, and taste. From bacteria to man, food detection and acquisition are fundamental to survival. All the senses are required to make decisions about ingestion, and TRP channels, abundantly present in the nerve endings of the mouth, tongue, and nose, play an important role in chemical sensing [1,2]. In the OE, TRPC1 and 6, TRPM3, 4, 5, 6, and 7, TRPV2 and 6, and TRPA1 have been shown to be expressed by RT-PCR [4]. Expression of TRPV1, 2, 3, and 4, and TRPM5 has also been demonstrated by immunohistochemistry [3,6].

The present study revealed immunoreactivity by TRPV1, 2, 3, and 4, TRPM5 and 8, and TRPA1 in murine OE, which confirms those previous investigations [3,4,6]. As regards the functional role of TRPVs, it has been suggested that TRPV1 may be involved in regulating cyclic nucleotide-gated channels and in short-term adaptation of the olfactory system [3]. Our finding of the presence of TRPV1 and TRPV2 in the ORNs, as well as in nerve fibers in the lamina propria, suggests that these TRP channels play a vital part in chemosensation by OE. Furthermore, TRPV1 is known to be expressed in the nasal trigeminal neurons and showed sensitivity to capsaicin [7]. Taken together, all these findings indicate that TRPV1 and TRPV2 play an important role in the peripheral and central olfactory/trigeminal interaction in nasal chemoreception. TRPV3 may underlie the mechanism of enhanced oral and nasal sensitization to successive exposures to certain odors, flavors, and irritants [8]. Consequently, it has been suggested that TRPV3 acts as a chemoesthetic receptor and is also involved in allergic rhinitis caused by repeated exposures to certain odors. TRPV4 is activated by heat and extracellular hypotonicity. Hypotonicity-induced activation of TRPV4 requires its interaction with the water channel aquaporin (AQP) 5 [9]. It has been reported that the response of the frog's olfactory system to water is elicited by reduced osmotic pressure [10]. In addition, an extensive network of AQP, including AQP5 expression, is present in the OE [11]. These findings indicate that TRPV4 is directly involved in fluid homeostasis in the OE.

TRPA1 is the only TRPA protein present in humans and other mammals. TRPA1 is activated chemically by the psychoactive component in marijuana, by environmental irritants, and by pungent compounds. These include ingredients present in wasabi, horseradish, and mustard oils (isothiocyanates); garlic (allicin); cinnamon oil (cinnamaldehyde); marijuana (tetrahy-drocannabinol); and tear gas (acrolein). TRPA1 can also be activated by stimulating the PLC pathway with bradykinin and the subsequently produced metabolites DAG and PUFAs [1,9].

It has not been conclusively established whether TRPA1 is a thermally or a mechanically gated channel. When exogenously expressed in cultured cells, TRPA1 has been reported to be a channel activated by injurious cold (< 17°C), although such activation in both cultured cells and TRPA 1-deficient mice is controversial. TRPA1 channels in mouse and zebra fish have been suggested to be mechanically gated channels, and the multiple ankyrin repeats of TRPA 1 may constitute a gating spring capable of transducing mechanical force and thereby facilitating channel opening. However, analyses of TRPA 1-deficient mice have refuted this possibility [1,9].

The present investigation has demonstrated that immunoreactivity to TRPA1 occurred in the ORNs, while the nerve fibers in the lamina propria showed weak immunoreactivity. TRPA1 is reportedly present in neurons of dorsal root ganglia (DRG) and trigeminal ganglia (TG) [1,9]. TRPA1 immunoreactivity was found in unmyelinated nerve fibers in the urothelium, in the suburothelial space, and in the muscle layer, as well as around blood vessels throughout the bladder [12]. All TRPA1 immunore-active nerve fibers also expressed TRPV1 immunore-activity and vice versa. TRPA1 was also detected in urothelial cells at both the transcriptional and the protein level. The presence of TRPA1 on C-fiber bladder afferents and in urothelial cells, and the finding that intravesical TRPA1 activators initiate detrusor hyperactivity, together indicate that TRPA1 participates in sensory transduction in this organ [12]. Our present findings mean that TRPA1 is also involved in chemosensation at the ORN level.

TRPM4 and TRPM5 are unusual in the TRP subfamily in that they are voltage-modulated, Ca^2+^-activated, monovalent cation selective channels. The monovalent selectivity, which sets these channels apart from other TRP channels, is warranted by a short acidic sequence of six amino acids in its pore loop. TRPM5 is also temperature-sensitive, being activated by heat in the range of 15–35°C. It is thus another example of a TRP channel that integrates thermal input with other modes of activation [1,9]. TRPM5 protein has been demonstrated in intestine, liver, lung, and taste bud cells [1,9]. Recently, TRPM5 was shown to be enriched in taste receptor cells, where it is essential for the perception of sweet, bitter, and umami taste compounds. Two independently generated TRPM5 knockout mouse models display diminished sweet, bitter, and umami perception. Interestingly, the sensitivity of TRPM5 to temperature was suggested to be the molecular mechanism underlying the psychophysical phenomenon of ‘thermal taste', i.e. enhanced sweetness perception with increasing temperature. TRPM5 is highly abundant in rodent chemosensory organs including OE and the vomeronasal organ (VNO), as well as in a subset of solitary cells distributed throughout the epithelia of the respiratory system and the gastrointestinal tract [13]. In the main OE, TRPM5 was detected in solitary epithelial cells. Occasionally, like typical ciliated olfactory cells, TRPM5-expressing cells have elongated cell bodies that reach both the lumen and the basal membrane. Furthermore, TRPM5 was detected in sensory epithelia of the VNO [13], in the apical part of the cell, and in the present study, also in the ORNs, especially on the apical surface. These data suggest that TRPM5 might play an as yet unappreciated physiological role in olfaction of odorants and pheromones.

TRPM8 is a thermally regulated channel activated by moderately cool temperatures (<23-28°C) and by agents that evoke a sensation of coolness, such as menthol, eucalyptol, and icilin. Activation by cold and menthol can be separated, as several mutations that have a profound effect on activation by menthol have only a minimal effect on activation by coolness. This shows that the domains involved in activation by menthol and thermal input are distinct. In further support of this conclusion, modulation of TRPM8 activity by pH has differential effects on activation by icilin and cold, versus menthol. The mechanisms for thermal activation of TRPV1 and TRPM8 by hot and cool temperatures, respectively, appear to be similar. These channels are voltage dependent, and their respective activation temperatures as well as ligands lead to shifts in their voltage thresholds toward more physiological membrane potentials [1,9]. TRPM8 protein has been demonstrated in DRG, TG, prostate, and liver [1,9]. The present study showed that TRPM8 was expressed mainly in the ORNs. These findings strongly indicate that TRPM8 is an essential factor in chemosensation in the OE and is probably related to the direct temperature reaction of the olfactory system to cool temperatures.

It has been demonstrated that at intermediate odor-ant concentrations, heat invariably causes a reduction in olfactory sensitivity, as they would be expected to compensate for the increase in volatiles in the air. Cold has the opposite effect [5]. These findings may indicate the existence of thermosensitive channels in the OE. Numerous mammalian TRP channels (thermo-TRPs), activated by temperature changes, account for a large proportion of the temperature range to which mammals respond. TRPV1 and TRPV2 are sensors for uncomfortably warm (>43°C) and very hot (>52°C) temperatures, respectively, whereas TRPV3 (>30-39°C) and TRPV4 (25-34°C) contribute to the perception of moderate temperatures. TRPM8 appears to function at cool temperatures, and TRPA1 may be a cold sensor [1]. Thermo-TRPs are present in neurons, such as those in the DRG or TG, which are known to function in thermo-sensation. Sensory neurons derived from TRPM8-null mice lack detectable levels of TRPM8 mRNA and protein and their number responding to cold (18°C) and menthol (100 *mM)* is greatly reduced [14]. These thermo-TRPs (TRPV1-4, TRPM8, and TRPA1) were all found in OE. The basis for previous and present findings is that these TRPs may function as thermosensitive calcium channels in the OE.

Several in vivo experimental models have been established for inducing mass degeneration of the ORNs. These include olfactory bulbectomy, olfactory nerve transection, topical application of chemicals such as zinc sulfate, Triton X-100, and methyl bromide into the nasal cavity, and systemic injection of nasotoxic drugs such as 3-methylindole, dichlobenil, and methimazole. The mode of cell death in the ORNs differs among these experimental models. Olfactory bulbectomy and olfactory nerve transection, which resemble olfactory dysfunction caused by head injuries, have been found to induce massive apoptosis in the mature ORN population. In contrast, when applied to ORNs most chemicals that potentially resemble toxic environmental stimuli have been reported to cause necrosis. Regarding methimazole-induced cell death, chiefly in the mature ORNs, the affected cells were TUNEL-positive and showed a nuclear staining pattern, which strongly suggests that such cell death in ORNs is predominantly due to apoptosis [15].

The present study revealed that the fluorescence intensity of TRPs of murine ORNs was weakened by methimazole treatment. These effects were sustained until 3 months after the treatment, indicating that the recovery of TRP expression takes a long time, which might explain the clinical condition that recovery of the sense of smell, lost due to head injury, is also a lengthy procedure. The other interesting results were that expression of TRPM8 was not reduced following methimazole treatment. Moreover, expression of TRPV1 and TRPV2 recovered earlier. In the clinical situation of patients with olfactory disturbance, menthol and curry odors are frequently identified, but Japanese orange, wood, and condensed milk odors, seldom, since menthol odorants stimulate not only the olfactory nervous system but also the trigeminal nerve system [13]. As has been noted, TRPM8 is a thermally regulated channel activated by moderately cool temperatures (<23–28°C) and by compounds that evoke a sensation of coolness, such as menthol, eucalyptol, and icilin. TRPV1 is activated by capsaicin and resinifer-atoxin, heat, H^+^, and encocannabinoid [1,9]. Expression of both TRPV1 and TRPM8 was demonstrated in the TG [13]. It is plausible that menthol and/or curry odors stimulate the trigeminal nerve system, although the present study found unreduced expression of TRPM8 or early recovery of TRPV1 in the ORNs. These findings may indicate another possibility - that unchanged or early recovery of TRPV1 and TRPM8 in the ORNs is closely related to the fact that patients with olfactory disturbance display a high rate of recognition of menthol and curry odors. The early recovery of TRPV2 is another interesting finding. It has been suggested that TRPV2 is important in the regeneration of ORNs by stimulating nerve growth factors [17]. The early recovery of TRPV2 expression observed in our study seems to support this hypothesis.

In conclusion, our study has identified TRPV1, 2, 3, and 4, TRPM5 and 8, and TRPA1 in the mouse OE, where their presence seems to play a vital role, contributing to olfactory chemosensation, olfactory adaptation, olfactory/trigeminal interaction, and OE fluid homeostasis. In the pathological condition, the expression of TRPV3, TRPV4, TRPM5, and TRPA1 was markedly reduced and recovery took a long time. In contrast, expression of TRPM8 was only minimally affected even in the pathological condition, while TRPV1 and TRPV2 showed early recovery after methimazole treatment.
